# Supplementary material for: Patterns and Predictors of Sitting among Women from Disad-Vantaged Neighbourhoods over Time: A 5-Year Prospective Cohort Study
Source: Int J Environ Res Public Health. 2021 Apr 27;18(9):4625. doi: 10.3390/ijerph18094625 (PMC8123820; doi:10.3390/ijerph18094625)
Supplement: Supplementary file 1 [file ijerph-18-04625-s001.zip › ijerph-1153342-supplementary.pdf]

Supplementary Table S1. Comparison of baseline characteristics between participants who completed surveys at three time points (T1, T2 and T3), participants who completed a survey only at T1, and participants who completed surveys at both T1 and T2

Variables	Completed surveys at T1, T2, T3 (n=1560)	Completed survey at T1 only (n=2347)	Completed surveys at T1 and T2 only
Age in years, (mean, SD)	36.2 (7.6)	33.1 (8.3)	33.7 (8.2)
BMI kg/m^2 (mean, SD)	26.1 (5.8)	26.0 (6.1)	26.1 (6.2)
Area of residence (N, %)			
Urban	623 (40.2)	1251 (51.7)	142 (40.3)
Rural	937 (59.8)	1155 (48.3)	211 (59.6)
General Health (N, %)			
Excellent	180 (12.8)	173 (7.5)	27 (7.9)
Very good	554 (37.9)	803 (34.7)	99 (29.3)
Good	599 (36.7)	968 (41.9)	155 (45.7)
Poor/Fair	177 (12.6)	369 (15.9)	58 (17.1)
Smoking status (N, %)			
Never smoked	817 (50.4)	1212 (49.8)	155 (49.0)
Used to smoke	414 (23.4)	558 (22.9)	94 (23.4)
Smoke occasionally	122 (10.1)	242 (9.9)	47 (10.3)
Smoke regularly	207 (17.1)	421 (17.4)	57 (17.3)
Level of Education (N, %)			
Low	331 (21.3)	534 (21.7)	81 (21.8)
Medium	755 (48.6)	1270 (53.8)	191 (53.8)
High	467 (30.1)	576 (24.5)	77 (24.3)
Employment status (N, %)			
Working full-time	578(37.7)	919 (39.8)	116 (33.4)
Working part-time	501 (32.7)	628 (26.5)	116 (33.4)
Not working	455 (29.6)	802 (33.7)	115 (33.2)
Average gross income (N, %)			
No income	112 (7.5)	164 (0.6)	31 (8.8)
$1-699 AUD/week	589 (39.0)	1293 (56.8)	193 (52.7)
$700-1499 AUD/week	616 (41.5)	454 (19.8)	69 (19.5)
$1500 AUD or more/week	46 (3.1)	46 (2.0)	9 (2.0)
Don't know/want to answer	133 (8.9)	323 (18.2)	32 (9.0)
Marital Status (N, %)			
Married	1128 (72.3)	1460 (60.4)	241 (68.5)
Separated	117 (7.8)	216 (8.8)	33 (9.4)
Never Married	296 (19.8)	735 (30.7)	78 (22.2)
Number of children (N, %)			
None	540 (36.5)	1006 (42.5)	110 (31.5)
One	264 (17.8)	431 (18.1)	78 (22.4)
Two	394 (26.6)	572 (24.2)	98 (28.1)
Three or more	281 (19.0)	364 (15.2)	63 (18.0)

Abbreviations: BMI- body mass index, SD- standard deviation, N (%)- number(percentage), T1=2007-08, T2=2010-11, T3=2012-13

Supplementary Table S2. Change in sitting over five years by sociodemographic and health characteristics: Complete case sensitivity analysis

		Unadjusted model			Adjusted model		
**Variables**		β-coefficient (95%CI)		p-value	β-coefficient (95% CI)		p-value
Age category (years)	Baseline difference						
	18-24years	Ref			Ref		
	25-34years	-1.3	(-3.4, 0.8)	0.219	-0.0	(-2.2, 2.1)	0.988
	35-45years	-4.5	(-6.2, -2.3)	<0.001	-1.1	(-3.2, -1.0)	0.311
	Average change per year	-0.4	(-1.0, 0.2)	0.224	-0.2	(-0.8, 0.4)	0.528
	18-24years						
	25-34years	-0.2	(-0.9, 0.5)	0.568	-0.4	(-1.2, 0.3)	0.270
	35-45years	0.6	(-0.1, 1.2)	0.105	0.4	(-0.3, 1.1)	0.320
BMI (kg/m^{2})	Baseline difference						
	Normal	Ref			Ref		
	Overweight (26-30)	1.8	(0.1, 3.5)	0.038	2.0	(0.3, 3.7)	0.020
	Obese (>30)	3.4	(1.6, 5.2)	<0.001	3.7	(1.8, 5.5)	<0.001
	Average change per year	0.0	(-0.3, 0.3)	0.921	0.1	(-0.2, 0.4)	0.528
	Normal						
	Overweight	-0.3	(-0.8, 0.3)	0.391	-0.3	(-0.8, 0.2)	0.243
	Obese (>30)	-0.4	(-0.9, 0.2)	0.174	-0.5	(-1.0, 0.1)	0.126
Area of Residence	Baseline difference						
	Urban	Ref			Ref		
	Rural	-5.1	(-6.4, -3.7)	<0.001	-3.7	(-5.1, -2.2)	<0.001
	Average change per year	-0.2	(-0.5, 0.1)	0.167	-0.3	(-0.6, 0.1)	0.09
	Urban						
	Rural	0.2	(-0.2, 0.7)	0.315	0.3	(-0.1, 0.8)	0.124
General Health	Baseline difference						
	Excellent	Ref			Ref		
	Very Good	1.7	(-0.7, 4.3)	0.168	1.4	(-1.7, 3.3)	0.274
	Good	2.8	(0.3, 5.3)	0.027	1.9	(-1.4, 3.5)	0.139

	Poor/fair	4.5	(1.6, 7.4)	0.002	2.2	(-1.4, 4.5)	0.155
	Average change per year	-0.1	(-0.8, 0.5)	0.676	-0.0	(-0.5, 0.7)	0.906
	Excellent						
	Very Good	0.1	(-0.7, 0.8)	0.835	0.0	(-0.7, 0.8)	0.917
	Good	-0.2	(-0.9, 0.5)	0.587	-0.4	(-1.1, 0.3)	0.306
	Poor/fair	0.4	(-0.5, 1.3)	0.361	0.1	(-0.8, 1.1)	0.743
Smoking status	Baseline difference						
	No smoking	Ref			Ref		
	Used to smoke	0.0	(-1.6, 1.7)	0.956	0.9	(-0.8, 2.5)	0.215
	Smoke Occasionally	0.1	(-2.3, 2.5)	0.958	-0.4	(-2.8, 2.2)	0.775
	Smoke regularly	1.1	(-0.8, 3.1)	0.256	1.6	(-0.4, 3.7)	0.121
	Average change per year	-0.1	(-0.4, 0.2)	0.410	-0.0	(-0.3, 0.3)	0.906
	No smoking						
	Used to smoke	0.0	(-0.5, 0.5)	0.918	-0.0	(-0.5, 0.5)	0.982
	Smoke Occasionally	0.3	(-0.5, 1.1)	0.407	0.2	(-0.6, 1.1)	0.682
	Smoke regularly	-0.5	(-1.1, 0.2)	0.170	-0.6	(-1.3, 0.1)	0.096
Education status	Baseline difference						
	Low	Ref			Ref		
	Medium	2.0	(0.2, 3.8)	0.025	-0.1	(-1.0, 1.7)	0.935
	High	2.4	(0.4, 4.3)	0.017	-0.4	(-2.5, 1.7)	0.698
	Average change per year	0.0	(-0.4, 0.5)	0.877	-0.1	(-0.6, 0.4)	0.756
	Low						
	Medium	-0.3	(-0.9, 0.2)	0.271	-0.9	(-0.7, 0.5)	0.767
	High	-0.1	(-0.7, 0.5)	0.743	0.1	(-0.5, 0.8)	0.633
Employment status	Baseline difference						
	Full time	Ref			Ref		
	Working part-time	-6.4	(-8.1, -4.8)	<0.001	-5.1	(-6.7, -3.4)	<0.001
	Not working	-8.5	(-10.1, -6.8)	<0.001	-7.6	(-9.2, -6.0)	<0.001
	Average change per year	-0.4	(-0.8, -0.1)	0.019	-0.3	(-0.7, 0.0)	0.051
	Full-time						
	Working part-time	0.3	(-0.1, 0.9)	0.077	0.4	(-0.0, 1.0)	0.146
	Not working	0.5	(-0.0, 1.0)	0.053	0.5	(0.0, 1.0)	0.049

Average gross income	Baseline difference						
	Nil	Ref			Ref		
	$1-699 AUD	3.6	(0.5, 5.3)	0.007	0.8	(-1.6, 3.3)	0.503
	$700-1499 AUD	9.2	(6.7, 12.0)	<0.001	4.6	(1.7, 7.6)	0.002
	$1500 AUD or more/week	17.2	(12.3, 22.1)	<0.001	14.4	(9.2, 19.5)	<0.001
	Don’t know/want to answer	3.9	(0.2, 6.2)	0.016	0.5	(-2.5, 3.5)	0.736
	Average change per year	0.0	(-0.9, 0.4)	0.989	0.0	(-0.7, 0.7)	0.960
	Nil						
	$1-699 AUD	0.1	(-0.4, 1.1)	0.982	0.1	(-0.6, 0.8)	0.771
	$700-1499 AUD	-0.4	(-1.0, 0.6)	0.351	-0.4	(-1.2, 0.3)	0.267
	$1500 AUD or more/week	-1.5	(-2.9, 0.0)	0.061	-1.2	(-2.8, 0.3)	0.113
	Don’t know/want to answer	-0.8	(-1.4, 0.6)	0.151	-0.7	(-1.7, 0.3)	0.186
Marital status	Baseline difference						
	Married	Ref			Ref		
	Separated/Divorced/Widowed	0.1	(-2.3, 2.6)	0.919	-0.3	(-2.7, 2.1)	0.800
	Never married	7.4	(5.8, 9.0)	<0.001	4.9	(3.1, 6.7)	<0.001
	Average change per year	-0.1	(-0.4, 0.1)	0.384	-0.1	(-0.3, 0.2)	0.486
	Married						
	Separated/Divorced/Widowed	0.4	(-0.4, 1.2)	0.291	0.4	(-0.4, 1.2)	0.338
	Never married	-0.1	(-0.6, 0.4)	0.694	-0.2	(-0.8, 0.3)	0.400
Number of children	Baseline difference						
	No children	Ref			Ref		
	One	-6.8	(-8.7, -4.9)	<0.001	-4.0	(-6.0, -2.0)	<0.001
	Two	-9.2	(-10.9, -7.5)	<0.001	-5.8	(-7.7, -3.9)	<0.001
	Three or more	-11.9	(-13.8, -9.9)	<0.001	-7.7	(-9.8, -5.5)	<0.001
	Average change per year	-0.8	(-1.1, -0.4)	<0.001	-0.7	(-1.1, -0.4)	0.001
	No children						
	One	0.5	(-0.1, 1.1)	0.103	0.6	(-0.1, 1.1)	0.091
	Two	0.9	(0.4, 1.5)	0.001	0.9	(0.4, 1.5)	<0.001
	Three or more	1.5	(0.9 ,2.2)	<0.001	1.5	(0.9, 2.1)	<0.001

Abbreviations: Ref- reference, BMI- body mass index, CI- confidence interval.
Each model adjusted:
Age category for BMI, area of residence and number of children.

BMI category for age, area of residence, health status, education, employment, marital status and number of kids.
Area of residence for BMI, marital status and number of children.
Health status for age, BMI, area of residence, education, employment, marital status and number of children.
Smoking status for age, BMI, area of residence, education, health status, marital status and number of children.
Employment status for area of residence and marital status.
Education status for BMI, area of residence, employment status, health status, marital status and number of children.
Income status for age, BMI, health status, marital status and number of children.
Marital status for age, area of residence and number of children.
Number of children for area of residence, employment status and marital status

Supplementary Table S3. Change in sitting over five years by sociodemographic and health characteristics: Sensitivity analysis truncating high/implausible sitting values

		Unadjusted model			Adjusted model		
**Variables**		β-coefficient (95%CI)		p-value	β-coefficient (95% CI)		p-value
Age category (years)	Baseline difference						
	18-24years	Ref			Ref		
	25-34years	-1.6	(-3.7, 0.5)	0.142	0.1	(-2.2, 2.2)	0.979
	35-45years	-5.0	(-6.9, -3.1)	<0.001	-1.2	(-3.3, 1.0)	0.296
	Average change per year	-0.7	(-1.3, -0.1)	0.026	-0.6	(-1.2, 0.0)	0.037
	18-24years						
	25-34years	-0.2	(-0.9, 0.5)	0.547	-0.1	(-0.9, 0.6)	0.680
	35-45years	0.7	(0.0, 1.4)	0.038	0.7	(0.0, 1.3)	0.047
BMI (kg/m$^{2)}$	Baseline difference						
	Normal	Ref			Ref		
	Overweight (26-30)	2.0	(0.4, 3.6)	0.014	2.4	(0.7, 4.1)	0.002
	Obese (>30)	2.9	(1.2, 4.6)	0.004	2.8	(1.0, 4.7)	0.005
	Average change per year	-0.2	(-0.5, 0.1)	0.158	-0.1	(-0.4, 0.2)	0.443
	Normal						
	Overweight	-0.4	(-0.9, 0.1)	0.95	-0.5	(-0.9, 0.3)	0.069
	Obese (>30)	-0.2	(-0.7, 0.4)	0.558	-0.2	(-0.7,0.4)	0.500
Area of Residence	Baseline difference						
	Urban	Ref			Ref		
	Rural	-5.8	(-7.1, -4.4)	0.001	-3.9	(-5.3, -2.6)	<0.001
	Average change per year	-0.6	(-0.9, -0.2)	<0.001	-0.5	(-0.9, -0.2)	0.001
	Urban						
	Rural	0.5	(0.0, 0.9)	0.030	0.5	(0.1, 0.9)	0.026
General Health	Baseline difference						
	Excellent	Ref			Ref		
	Very Good	0.6	(-1.9, 3.2)	0.643	-0.3	(-2.8, 2.2)	0.797

	Good	1.7	(-0.8, 4.2)	0.193	0.1	(-2.4, 2.6)	0.734
	Poor/fair	4.2	(1.2, 7.1)	0.006	1.7	(-1.2, 4.7)	0.247
	Average change per year	-0.5	(-1.1, 0.1)	0.118	-0.4	(-1.0, 0.3)	0.264
	Excellent						
	Very Good	0.3	(-0.5, 0.9)	0.540	0.2	(-0.5, 0.9)	0.604
	Good	0.1	(-0.7, 0.7)	0.948	0.1	(-0.8, 0.7)	0.927
	Poor/fair	0.5	(-0.3, 1.4)	0.217	0.4	(-0.5, 1.3)	0.359
Smoking status	Baseline difference						
	No smoking	Ref			Ref		
	Used to smoke	-1.1	(-2.8, 0.6)	0.207	-0.0	(-1.7, 1.6)	0.406
	Smoke Occasionally	0.2	(-2.3, 2.7)	0.872	-0.8	(-3.2, 1.6)	0.497
	Smoke regularly	-0.1	(-2.1, 1.9)	0.914	-0.2	(-2.1, 1.8)	0.568
	Average change per year	-0.5	(-0.7, -0.1)	0.003	0.3	(-0.6, 0.0)	0.032
	No smoking						
	Used to smoke	0.3	(-0.2, 0.8)	0.294	0.2	(-0.3, 0.7)	0.406
	Smoke Occasionally	0.3	(-0.4, 1.1)	0.394	0.3	(-0.5, 1.1)	0.407
	Smoke regularly	-0.1	(-0.7, 0.5)	0.773	-0.2	(-0.8, 0.4)	0.457
Education status	Baseline difference						
	Low	Ref			Ref		
	Medium	2.3	(0.5, 4.1)	0.009	0.1	(-1.7, 1.9)	0.795
	High	4.0	(2.0, 6.1)	<0.001	1.1	(-0.9, 3.2)	0.345
	Average change per year	0.1	(-0.4, 0.5)	0.873	-0.1	(-0.5, 0.4)	0.795
	Low						
	Medium	-0.5	(-0.9, 0.1)	0.097	-0.3	(-0.8, 0.3)	0.349
	High	-0.6	(-1.2, -0.1)	0.038	-0.4	(-1.0, 0.2)	0.194
Employment status	Baseline difference						
	Full time	Ref			Ref		
	Working part-time	-9.4	(-11.1, -7.7)	<0.001	-6.8	(-8.5, -5.1)	<0.001
	Not working	-10.5	(-12.2, -8.9)	<0.001	-7.8	(-9.6, -6.1)	<0.001
	Average change per year	-1.1	(-1.4, -0.7)	<0.001	-0.9	(-1.3, -0.6)	<0.001
	Full-time						
	Working part-time	1.1	(0.6, 1.6)	<0.001	1.1	(0.6, 1.6)	<0.001

	Not working	1.2	(0.6, 1.7)	<0.001	1.2	(0.7, 1.7)	<0.001
Average gross income	Baseline difference						
	Nil	Ref			Ref		
	$1-699 AUD	2.8	(0.1 ,5.6)	0.043	0.6	(-2.1, 3.4)	0.643
	$700-1499 AUD	11.9	(8.9, 14.3)	<0.001	6.7	(3.6, 9.9)	<0.001
	$1500 AUD or more/week						
	Don't know/want to answer	3.8	(0.5, 7.1)	0.023	0.9	(-2.3, 4.3)	0.557
	Average change per year	-0.4	(-1.1, 0.3)	0.287	-0.1	(-0.7, 0.6)	0.897
	Nil						
	$1-699 AUD	0.3	(-0.4, 1.0)	0.413	0.1	(-0.6, 0.8)	0.774
	$700-1499 AUD	-0.8	(-1.6, -0.1)	0.042	-1.0	(-1.8, -0.3)	0.009
	$1500 AUD or more/week	-1.7	(-3.9, 0.1)	0.331	-1.3	(-2.9, 0.3)	0.213
	Don't know/want to answer	-0.6	(-1.6, 0.3)	0.199	-0.9	(-1.9, 0.1)	0.057
Marital status	Baseline difference						
	Married	Ref			Ref		
	Separated/Divorced/Widowed	-0.8	(-3.3, 1.7)	0.522	-1.4	(-3.9, 1.0)	0.258
	Never married	7.5	(5.9, 9.1)	<0.001	4.3	(2.5, 6.1)	<0.001
	Average change per year	-0.3	(-0.6, -0.1)	0.008	-0.3	(-0.5, -0.1)	0.025
	Married						
	Separated/Divorced/Widowed	0.7	(-0.1, 1.5)	0.068	0.6	(-0.4, 1.4)	0.108
	Never married	-0.1	(-0.6, 0.4)	0.734	-0.2	(-0.7, 0.3)	0.446
Number of children	Baseline difference						
	No children	Ref			Ref		
	One	-8.6	(-10.5, -6.6)	<0.001	-5.7	(-7.7, -3.6)	<0.001
	Two	-10.6	(-12.3, -8.8)	<0.001	-6.9	(-8.8, -4.9)	<0.001
	Three or more	-13.6	(-15.6, -11.6)	<0.001	-9.1	(-11.4, -6.9)	<0.001
	Average change per year	-1.1	(-1.5, -0.8)	<0.001	-1.1	(-1.4, -0.7)	<0.001
	No children						
	One	0.9	(0.3, 1.5)	0.005	0.9	(0.3, 1.5)	0.003
	Two	1.2	(0.6, 1.7)	<0.001	1.2	(0.7, 1.7)	<0.001
	Three or more	1.8	(1.2, 2.4)	<0.001	1.8	(1.2, 2.4)	<0.001

Abbreviations: Ref- reference, BMI- body mass index, CI- confidence interval.

Each model adjusted:
Age category for BMI, area of residence and number of children.
BMI category for age, area of residence, health status, education, employment, marital status and number of kids.
Area of residence for BMI, marital status and number of children.
Health status for age, BMI, area of residence, education, employment, marital status and number of children.
Smoking status for age, BMI, area of residence, education, health status, marital status and number of children.
Employment status for area of residence and marital status.
Education status for BMI, area of residence, employment status, health status, marital status and number of children.
Income status for age, BMI, health status, marital status and number of children.
Marital status for age, area of residence and number of children.
Number of children for area of residence, employment status and marital status
